# Aspiration of a drug in a blister pack

**DOI:** 10.1002/rcr2.492

**Published:** 2019-09-25

**Authors:** Masataka Mori, Kasumi Kusanagi, Shuhei Ashikari, Takashi Iwanami, Manabu Yasuda, Takeshi Hanagiri

**Affiliations:** ^1^ Department of Thoracic Surgery Shin Kokura Hospital, Federation of National Public Service, Personnel Mutual Aid Associations Kitakyushu Japan

**Keywords:** Airway foreign body, blister pack, flexible bronchoscopy, press‐though package

## Abstract

We report a rare case of aspiration of a drug in a press‐through package (PTP) treated by not just pulling it but using a unique technique. A 73‐year‐old woman was referred to our department because of a persistent cough resulting from aspiration of a PTP. Flexible bronchoscopy identified the PTP in the trachea immediately above the carina. Just pulling the centre of the PTP edge with biopsy forceps could not move it, and we then rotated it by pulling the corner of the PTP edge to directly below the vocal cord. Passing over the vocal cord was difficult, which made us remove the bronchoscope and urge the patient to cough. These rotation techniques and voluntary coughing successfully removed the foreign body. This unique procedure may aid in the removal of a similar foreign body using a flexible bronchoscope forceps with insufficient grasping force.

## Introduction

Press‐through packages (PTPs), called blister packs, are used to pack drugs individually. Occasionally, cases of accidental swallowing of PTPs are reported. However, the presence of PTPs as lower airway foreign bodies is extremely rare. Here, we report a case of aspiration of PTP treated with a unique technique.

## Case Report

**Figure 1:**
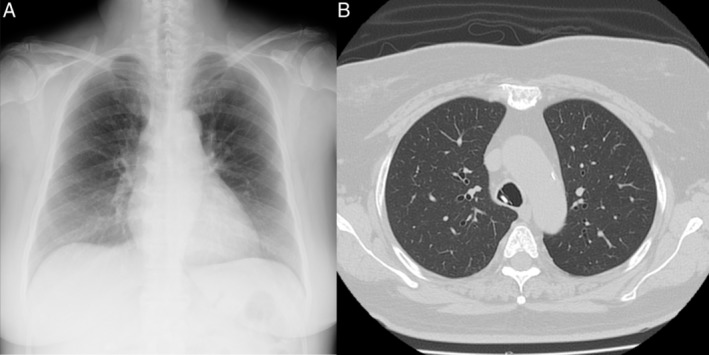
(A) Chest X‐ray showing no abnormal findings. (B) Computed tomography scan showing a foreign body in the trachea.

**Figure 2:**
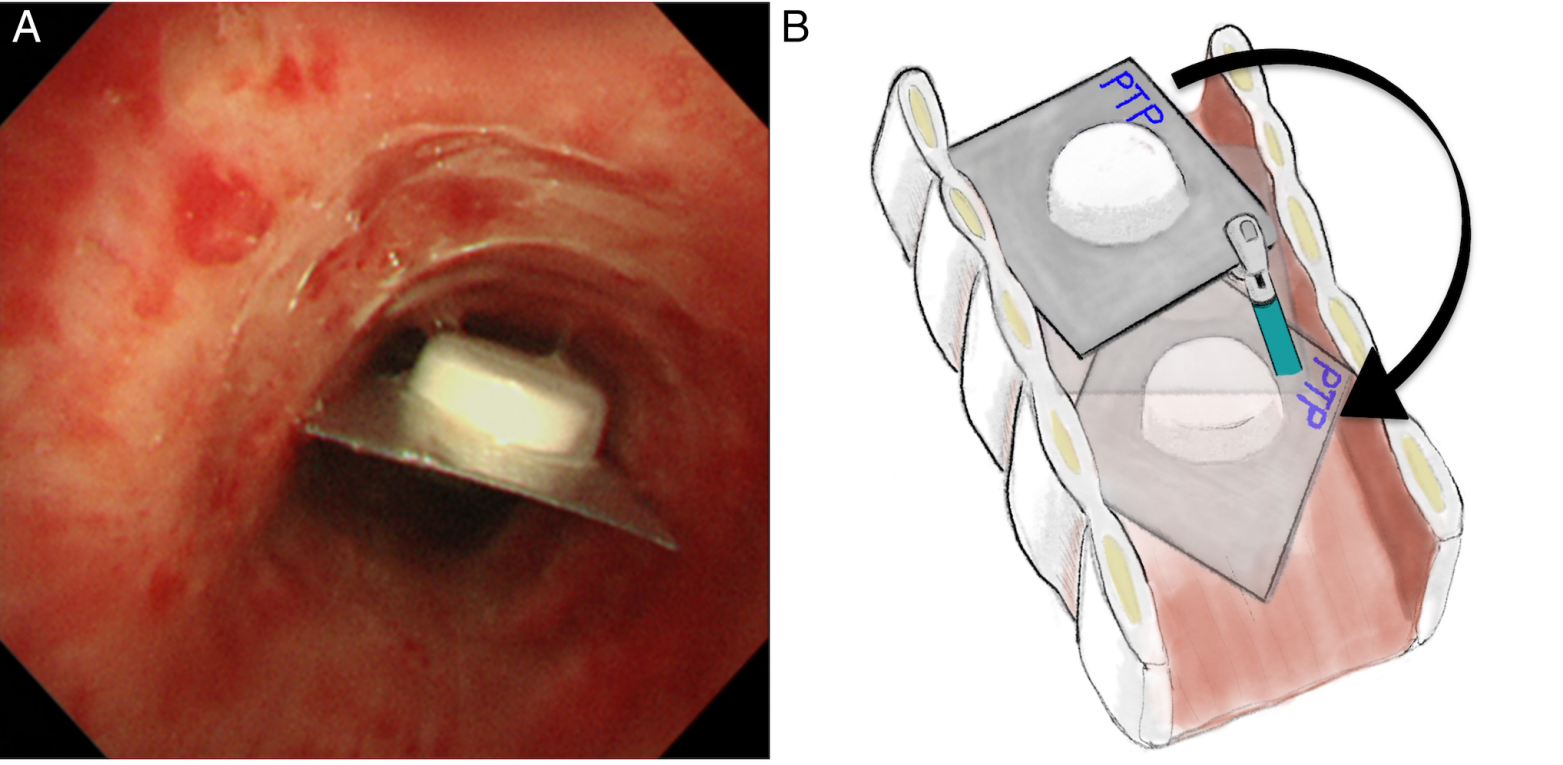
(A) Flexible bronchoscopy image demonstrating a press‐through package with a drug immediately above the carina in trachea. (B) The press‐through package began to rotate when the corner was pulled.

A 73‐year‐old woman was referred to our department because of a persistent cough without any haemoptysis since breakfast 3 h previously. No apparent abnormality was observed on the chest X‐ray (Fig. 1A). Computed tomography of the chest suggested the presence of a foreign body above the carina, shaped like a press‐through package with a maximal length of 20 mm (Fig. 1B). Flexible bronchoscopy was performed with application of topical anaesthesia (lidocaine) and administration of a sedative (midazolam). Using an Olympus bronchoscope 1T260, the foreign‐body PTP was located immediately above the main carina in the trachea (Fig. 2A). Although we tried to extract it by pulling the centre of the edge of the PTP with biopsy forceps, it did not move because both edges were caught on the tracheal wall. Unfortunately, alligator jaw grasping forceps that allows a greater grasping force than biopsy forceps were not available. We then rotated it carefully by pulling the corner of the edge (Fig. 2B). The PTP was pulled up to the subglottic space easily; however, it was very difficult to draw it out from the vocal folds. After several attempts, we removed the bronchoscope and urged the patient to cough; she successfully expectorated it herself. The PTP measured 21 × 17 × 3 mm with sharply demarcated edges. The patient was discharged in a stable condition with her voice intact a day after the operation.

## Discussion

Cases of accidental swallowing of PTPs have been reported previously 1, 2. The swallowed PTPs can cause perforations of the digestive tract 3, 4. In contrast, the presence of PTPs as airway foreign bodies is quite rare, and to the best of our knowledge, only one case of a PTP in the upper airway has been reported 5. Moreover, no cases of PTPs in the lower airway have been reported.

In our case, we were unsuccessful in pulling out the PTP by grasping the centre of the edge of the PTP and tugging it. The adhesive force between both the edges of the PTP and the tracheal wall was greater than the gripping force of the biopsy forceps. We were finally able to move the PTP by grasping and pulling its corner, thereby rotating it until it was directly below the vocal cord. To pass over the vocal cord, the shape of the PTP must precisely fit the shape of the slit of the vocal cord; hence, it was difficult to remove the PTP only by bronchoscopy. After removal of the bronchoscope, the patient was able to cough up the PTP successfully. Fortunately, the PTP did not injure the vocal cord or become caught in the glottis during expectoration. Self‐expectoration involves these risks, which might lead to dysphonia or respiratory failure; hence, if it is unavoidable, we have to provide a suitable explanation of the risks to the patient in advance and prepare emergency procedures such as tracheostomy and cricothyroidotomy. Furthermore, if general anaesthesia was used rather than topical anaesthesia, the removal of the PTP with the biopsy forceps alone would have been more challenging. An alternate approach to remove the foreign body would have been via a rigid bronchoscope and rigid grasping forceps. However, this was not available in our centre at the time of the procedure.

This case of a foreign body lodged in the lower airway suggests that the rotation technique described above and voluntary coughing may aid in the removal of a foreign body when using flexible bronchoscope forceps with insufficient grasping force.

### Disclosure Statement

Appropriate written informed consent was obtained for publication of this case report and accompanying images.
